# Monitoring Dicer‐Mediated miRNA‐21 Maturation and Ago2 Loading by a Dual‐Colour FIT PNA Probe Set

**DOI:** 10.1002/cbic.202000173

**Published:** 2020-05-26

**Authors:** Natalia Loibl, Christoph Arenz, Oliver Seitz

**Affiliations:** ^1^ Department of Chemistry Humbolt-Universität zu Berlin Brook-Taylor-Strase 2 12489 Berlin Germany

**Keywords:** Dicer, FIT PNA probes, miR21, pre-miR21, miRNA maturation

## Abstract

The inhibition of micro RNA (miRNA) maturation by Dicer and loading matured miRNAs into the RNA‐induced silencing complex (RISC) is envisioned as a modality for treatment of cancer. Existing methods for evaluating maturation either focus on the conversion of modified precursors or detect mature miRNA. Whereas the former is not applicable to native pre‐miRNA, the latter approach underestimates maturation when both nonmatured and matured miRNA molecules are subject to cleavage. We present a set of two orthogonally labelled FIT PNA probes that distinguish between cleaved pre‐miRNA and the mature miRNA duplex. The probes allow Dicer‐mediated miR21 maturation to be monitored and Ago2‐mediated unwinding of the miR21 duplex to be assayed. A two‐channel fluorescence readout enables measurement in real‐time without the need for specialized instrumentation or further enzyme mediated amplification.

## Graphical abstract

**Combined use** of two orthogonally labelled FIT PNA probes allows unambiguous monitoring of miRNA‐21 maturation. One fluorogenic hybridization probe signals the presence of intact pre‐miR21, the other probe shows enhanced fluorescence upon formation of the matured miRNA passenger strand. PNA‐FIT probes also provide a read‐out for monitoring the Ago2‐mediated unwinding of the miR21 duplex in real‐time.

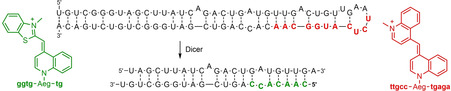

## Introduction

Micro‐RNAs (miRNAs) are small noncoding RNA molecules (∼22 nucleotides), which participate in the regulation of gene expression of essential cell processes such as proliferation, differentiation, apoptosis and others. Dysregulation of miRNA expression is associated with various diseases.^1^ For example, overexpression of oncogenic micro‐RNA 21 (miR21), which down‐regulates tumour suppressor genes, has been shown to cause cancer in a mouse model.^2^ Consequently, miRNA antagonization is a promising approach to combat cancer.^3^

Recent work suggests that small molecules are able to antagonize miRNA function by way of interference with miRNA maturation.^4^ Methods that allow monitoring maturation of specific miRNA molecules are required for screening campaigns and verification of the mechanism of action. The biogenesis of miRNA involves, after transcription, processing of the initially transcribed primary miRNA (pri‐miRNA) by the RNase Drosha. The stem‐looped precursor miRNA (pre‐miRNA) is transported to the cytoplasm, where cleavage by the RNase Dicer affords the matured miRNA duplex. Eventually, the miRNA strand is loaded into the RNA induced silencing complex (RISC) which binds to the target to accomplish its regulation mission.

Typical nucleic acid detection methods such as Northern blotting,^5^ real time‐polymerase chain reaction (RT‐PCR),^6^ RNAseq^7^ and microarrays^8^ have been applied to measurements of mature miRNAs. These methods require dedicated equipment and involve several, time‐consuming sample handling steps. Modern detection methods take advantage of isothermal amplification,^9^ which allow measurements in homogenous phase. However, the (bio)chemical steps leading to amplification prolong the assay time and it is, therefore, not feasible to monitor miRNA maturation in or near real time. Most of the reported methods focus on the detection of matured miRNA and little attention is paid to the detection of the non‐processed miRNA precursors.

An ideal assay should i) allow sensitive detection with standard equipment, ii) proceed in solution phase to enable rapid measurements near real time, iii) not require enzymes to allow – if required – the use of detergents and solvents and – if desired – enable monitoring within living cells and iv) allow simultaneous detection of both precursor and matured miRNA. In an early example, a pre‐miRNA was labelled with fluorescence donor and acceptor dyes.^10^ The probe showed increased donor emission upon Dicer‐mediated cleavage. However, this method requires modification of the pre‐miRNA and is, therefore, not applicable to natural miRNA precursors.

Herein we describe the development of a simple method for monitoring miR21 maturation on both levels: pre‐miRNA and miRNA. The method relies on a combination of two fluorogenic forced intercalation peptide nucleic acid probes (FIT PNA). FIT probes are hybridization probes, in which a canonical nucleobase is replaced by a dye of the thiazole orange family of intercalators.^11^ Upon hybridization with the complementary target, the dye intercalates between select base pairs of the formed probe‐target duplex.^12^ This leads to increases of fluorescence due to restriction of torsions around the methine bridge. Previously, we, and others, used PNA‐ and DNA‐based FIT probes in RT‐PCR,^13^ wash‐free RNA‐fluorescence in situ hybridization (RNA‐FISH)^14^ and RNA imaging in living cells.^15^ Herein, we designed the fluorogenic FIT PNA probes for monitoring Dicer‐mediated maturation of miR21. One probe signals the presence of the non‐matured pre‐miR21, while the second, spectrally distinct probe responds to formation of the matured miR21 strand and its incorporation into the RISC.

## Results and Discussion

**Figure 1:**
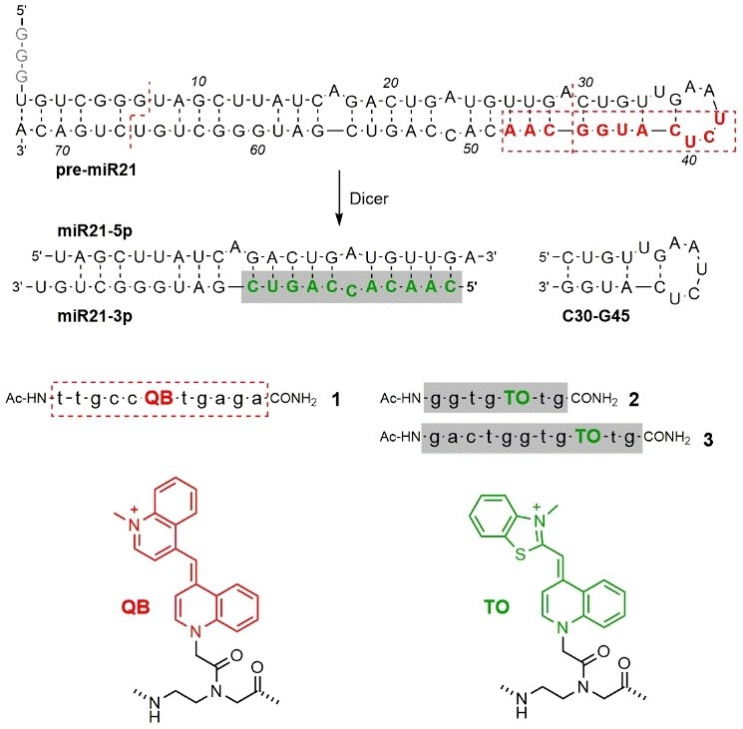
Dicer‐mediated cleavage of pre‐miR21 and FIT PNA probes **1**, **2** and **3** used for detection of both pre‐miR21 (**1**) and miR21‐3p (**2** and **3**). The 5′‐terminal G residues arise from the promoter used for synthesis by in vitro transcription.

During miR21 maturation Dicer cleaves the 72‐nt precursor miR21 (pre‐miR21) and liberates a duplex (matured miRNA) comprised of the strands miR21‐5p (miR21 from 5′ arm) and miR21‐3p (miR21 from 3′ arm) along with the 16‐nt hairpin segment (C30‐G45, Figure 1). For the design of a probe that is specific for pre‐miR21 we targeted the junction between miR21 and the loop part (red in pre‐miR21, Figure 1). Based on results of a reported SHAPE analysis^16^ and contrary to predictions by mfold, this stem‐loop region (C30 to G45) is highly dynamic and, therefore, accessible. The design of a probe that responds to the formation of the miR21‐5p•miR21‐3p duplex seemed more challenging given that the mature sequence is fully presented in both the pre‐miR21 and the matured structures. We assumed it will be preferable, if the probe targets a segment that overlaps with the sequence targeted by the pre‐miR21‐specific probe, which by means of competition could hinder a maturation product‐specific probe to bind pre‐miR21. We furthermore envisioned that opening of the double‐stranded (ds) region by strand invading PNA that is complementary to the C46‐C56 segment (green in Figure 1) would be easier for the miR21‐5p•miR21‐3p duplex than for the stem region of pre‐miR21. The latter duplex should be thermodynamically favoured due to the presence of the C30‐G45 hairpin.

**Figure 2:**
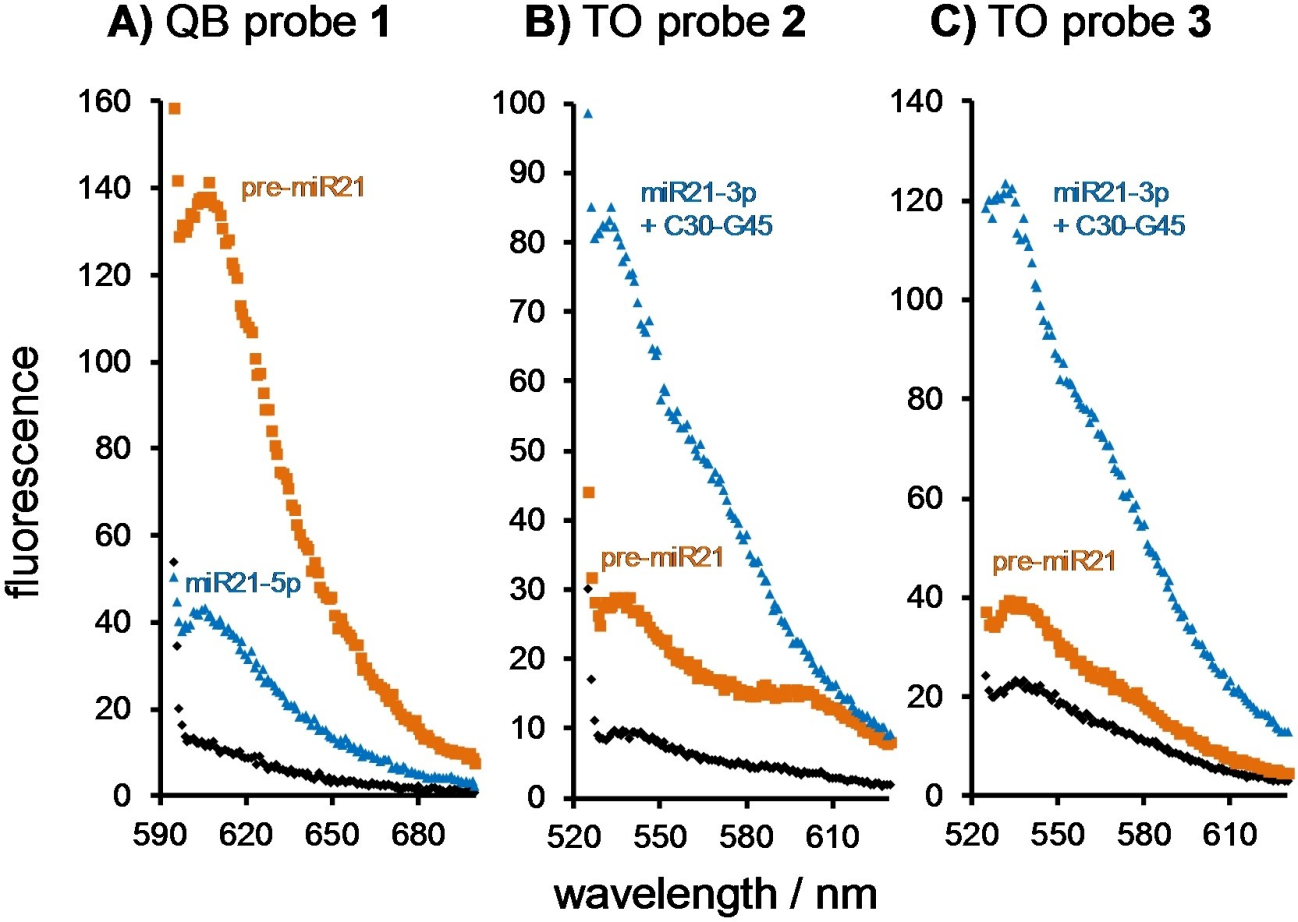
Fluorescence spectra of A) QB‐FIT PNA **1**, B) TO‐FIT PNA **2** and c) TO‐FIT PNA **3** as single strand (black), in presence of pre‐miR21 (orange) or in presence of A) miR21‐5p, miR21‐3p and C30‐G45 (blue) or B), C) miR21‐3p (blue). Conditions: 0.5 μM probes and targets in 10 mM NaH_2_PO_4_, 137 mM NaCl, 2.7 mM KCl, 3 mM MgCl_2_, pH 7.0, 37 °C, QB: *λ*_ex_=588 nm; TO: *λ*_ex_=516 nm.

The target sequence for the pre‐miRNA specific probe was designed to cover a Dicer cleavage site so that efficient binding of the probe should no longer take place after processing. The 11‐nt PNA strand **1** is complementary to the U38‐A48 segment of pre‐miR21. The red emitting quinoline blue (QB) dye15b, 15f was introduced at various positions (Table S1). This “QB‐walk” revealed FIT PNA **1** as a probe providing a 14‐fold enhancement of fluorescence upon addition of pre‐miR21 (Figure 2A, Table 1).

**Table 1:** Fluorescence enhancement of FIT PNA probes upon the addition of components of Dicer‐mediated miR21 maturation.

		*I*/*I*_0_^[a]^	
	QB probe **1**	TO probe **2**	TO probe **3**
pre‐miR21	13.7^[b]^ 12.7^[c]^	5.5^[b]^ 3.3^[c]^	/ 1.2^[c]^
miR21‐3p	/	9.5^[b]^ 10.3^[c]^	/ 4.4^[c]^
miR21‐5p+miR21‐3p+ C30‐G45 (product mix)	4.3^[c]^	4.2^[c]^	3.9^[c]^
product mix/ pre‐miR21	0.3^[c]^	1.3^[c]^	3.3^[c]^

[a] QB: *λ*_ex_=588 nm, *λ*_em_= 606 nm; TO: *λ*_ex_=516 nm, *λ*_em_=536 nm. Conditions: 0.5 μM probes and targets in 10 mM NaH_2_PO_4_, 137 mM NaCl, 2.7 mM KCl, pH 7.0, 37 °C [b] without or [c] with 3 mM MgCl_2_.

Processing of pre‐miR21 by Dicer leads to a product mix, consisting of the miR21‐5p•miR21‐3p duplex and the C30‐G45 segment, which is degraded by RNases in a cell. However, the latter would not occur in Dicer cleavage assays performed *ex cellulo*. To emulate such a scenario, the product mix consisting of miR21‐5p, miR21‐3p and the C30‐G45 segment was added to QB‐PNA probe **1**. The fourfold increase in fluorescence was substantially lower than the 14‐fold increase observed upon addition of the pre‐miR21 (Figure 2A; Table 1). Control experiments revealed that the comparably modest fourfold enhancement observed upon incubation with the product mix is due to recognition of the C30‐G45 component (Figure S1). We inferred: a strong (>fivefold) increase in QB emission from probe **1** should inform about the presence of intact pre‐miR21.

In the pursuit of a probe that provides a positive response upon processing of pre‐miR21, we first tested short, 7‐nt miR21‐3p specific PNA probes. We initially assumed that short PNA probes would better discriminate between the 22‐nt miR21‐3p and the 75‐nt pre‐miR21 stem‐loop hairpin, because the probe hybridization to the stem region of pre‐miR21 requires opening of the hairpin or, at least, unwinding of the duplex in the stem region. As the QB fluorophore was already used for the pre‐miR21 monitoring, we decided to use the thiazole orange (TO) dye for the detection of matured miR21. We evaluated four probes, which contained TO at different positions (Table S2). All four TO PNA probes showed a strong increase (6.2‐ to 19.6‐fold) of TO emission upon addition of miR21‐3p. Perhaps surprisingly, nearly equally high fluorescence responses (4.7‐ to 14.8‐fold) were observed when unprocessed pre‐miR21 was added. This unexpected result suggests that the structure of the pre‐miR21 hairpin is highly dynamic and readily opened. The most suitable TO probe **2** reported recognition of miR21‐3p by a 9.5‐fold enhancement of TO emission compared to a 5.5.‐fold upon addition of pre‐miR21 (Figure 2B, Table 1). We furthermore noticed that the presence of the miR21‐5p very effectively attenuated the response of **2** to miR21‐3p (Table 1, compare rows 2 and 3 for TO probe **2**).

To minimize the unwanted binding of probe **2** to pre‐miR21 and augment differences in the duplex stability between pre‐miR21 and miR21‐5p•miR21‐3p, we on the one hand stabilized duplex structures by including 3 mM MgCl_2_ in the assay buffer (Table 1, entries with superscript [c]), while on the other hand, we increased the length of TO FIT PNA probe to facilitate displacement of the miR21‐3p strand from the miR21‐5p•miR21‐3p duplex (Table S3). The longer probes showed a reduced responsiveness of the TO emission. For example, whereas the addition of pre‐miR21 to the heptamer probe **2** was accompanied by a 3.3‐fold enhancement of fluorescence, the fluorescence turn‐on was reduced to a 1.2‐fold with the 11‐nt TO‐PNA **3** (Figure 2C). Most importantly, the 11‐mer probe **3** still responded to miR21‐3p by affording a 4.4‐fold increase of fluorescence which was only slightly reduced to a 3.9‐fold in the presence of miR21 (compared to 10.3‐fold to 4.2‐fold (Table 1) with PNA‐heptamer **2**). In brief, the product mix provided a 3.3‐fold higher emission than the presence of the unmatured pre‐miR21.

**Figure 3:**
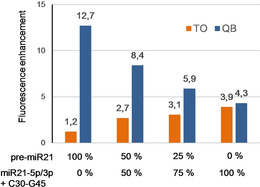
Fluorescence enhancement (*I*/*I*_0_) upon addition of pre‐miR21/miR21‐5p/miR21‐3p/C30‐G45 mixtures to a 1 : 1 mixture of QB‐FIT PNA probe **1** and TO‐FIT PNA probe **3**. Conditions: 0.5 μM probes and targets in 10 mM NaH_2_PO_4_, 137 mM NaCl, 2.7 mM KCl, 3 mM MgCl_2_, pH 7.0, 37 °C. TO: *λ*_ex_= 516 nm, *λ*_em_= 525–700 nm; QB: *λ*_ex_= 588 nm, *λ*_em_= 598–700 nm.

We concluded that the combination of QB probe **1** and TO probe **3** should allow unambiguous monitoring of Dicer‐mediated pre‐miR21 cleavage. The probe set was tested in a model of the dicer cleavage reaction, where pre‐miR21 was mixed in different proportions with the product mix comprised of miR21‐5p, miR21‐3p and the A30‐G45 segment (Figure 3). The emission enhancement provided by the QB‐FIT PNA probe gradually decreased from 12.7‐fold via 8.4‐fold to 4.3‐fold as the content of cleavage product was increased from 0 to 50 and eventually 100 %. At the same time, with increasing amounts of cleavage products, the enhancement of TO emission from **3** increased from 1.2‐ to 2.7‐fold and 3.9‐fold for 50 and 100 % cleavage products, respectively. Thus, the probe set successfully monitored the processing of the model Dicer reaction on both levels, pre‐miR21 and matured miR21, showing decreasing amount of pre‐miR21 and increasing amount of miR21 in the reaction mix. The ratio of the TO and QB emission enhancements changes by a factor of 11 (0 % cleavage: *I*(TO)×*I*_0_(QB)/*I*_0_(TO)×*I*(QB)=0.09; 100 % cleavage: *I*(TO)×*I*_0_(QB)/*I*_0_(TO)×*I*(QB)=0.91) providing a high dynamic range that should allow the detection pre‐miR21 processing when Dicer activity is low.

**Figure 4:**
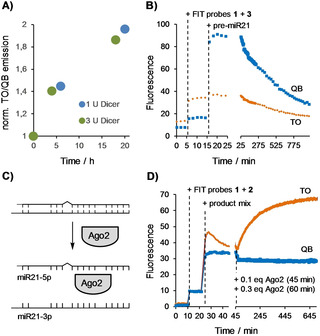
Cleavage of pre‐miR21 induced by A) 1 U or 3 U Dicer or B) 10 U Dicer per μg RNA is reported by monitoring A) TO/QB emission ratios or B) QB and TO emission from QB‐PNA FIT probe **1** and TO‐PNA FIT probe **3**. C) Ago2 loading induces the release of miR21‐3p. D) Ago2 loading increases emission from TO‐PNA FIT probe **2** but not of QB‐PNA FIT probe **1**. Conditions: A) 0.5 μM and B) 0.23 μM probes and pre‐miR21 and 1 U, 3 U or 10 U Dicer in 20 mM Tris, 12 mM NaCl, 2.5 mM MgCl_2_, 1 mM DTT, pH 7.7, 37 °C. D) 0.5 μM probes and 0.5 μM product mix (miR21‐5p, miR21‐3p, C30‐G45) and 0.4 equiv. Ago2 in 130 mM KCl, 30 mM Tris, 1.1 mM MgCl_2_, 1 mM DTT, 0.1 mM EDTA, pH 7.25. Settings for kinetic mode measurements: TO, *λ*_ex_=516 nm, *λ*_em_=536 nm; QB, *λ*_ex_=588 nm, *λ*_em_=606 nm.

Next, the probe set was used to characterize the cleavage of pre‐miR21 by recombinant human Dicer, which closely resembles native human Dicer. The enzyme (rhDicer, Genlantis) has a preference for dicing dsRNA templates of 500–1000 bp in length and cleavage yields typically are low if the dsRNA substrate is smaller than 300 bp. Indeed, after 20 h incubation with 1 U rhDicer per μg pre‐miR21, the ratio of TO/QB emission enhancements was increased by a factor of two (as opposed to tenfold expected for 100 % cleavage), indicative for a cleavage yield of 20 % (Figure 4A). Little change of cleavage was observed when the Dicer load was increased to 3 U per μg RNA. According to the manufacturer's protocol, an increase of the Dicer load is not recommended. Indeed, while the loss of QB emission from probe **1** indicates that pre‐miR21 is effectively cleaved at a tenfold increased load of Dicer (10 U per μg pre‐miR21), the concomitant decrease of emission from the TO probe **3** suggests that under these forcing conditions rhDicer induces also other cleavage reactions (Figure 4B). Dicer preferentially cleaves pre‐miRNA and, albeit at reduced rate, also dsRNA having 5′‐overhangs.^17^ Ando et al. reported that rhDicer can cleave pre‐miR21 obtained by in vitro transcription also between U26‐U27 and A48‐C49.^18^ This would destroy the binding site for TO probe **3** and explains the loss of TO emission from probe **3** upon treatment of pre‐miR21 with large amounts of rhDicer.

Protein argonaut‐2 (Ago2) is a crucial component of the RISC machinery. The protein is responsible for loading one of the miRNA strands into the RISC complex (Figure 4C). After Ago2‐loading, the guide miRNA directs the RISC complex to the complementary mRNA target for gene‐silencing. Recently, Ago2 loading has been proposed as a critical and druggable step in miRNA function.^19^ However, a simple, homogeneous assay is required to identify inhibitors by screening. We speculated that the highly responsive heptamer TO‐FIT PNA probe **2** could allow fluorescence monitoring of the Ago2‐mediated dissociation of the miR21‐5p•miR21‐3p duplex. The product mix comprised of miR21‐5p, miR21‐3p and the C30‐G45 segment was added to TO‐PNA‐heptamer **2** and the QB‐PNA‐11‐mer **1**. As expected, the probes responded by showing a fourfold increase in fluorescence. Of note, emission from TO probe **2** peaked directly after addition of the product mix and decreased within the next 20 min. We speculate that addition of **2** induces a partial opening of the miR21‐5p•miR21‐3p duplex followed by conformational rearrangements around the TO dye. Based on the previous experiments, loading of miR21‐5p onto Ago2 should increase TO emission due to facilitated access to miR21‐3p and formation of a **2**•miR21‐3p duplex whereas QB emission should remain unaffected. Indeed, addition of 0.4 equiv. Ago2 resulted in a gradual increase of TO emission (Figure 4D). Ago2 loading was a slow process, which required >10 h until a plateau was reached. Control experiments which also involved the use of heat‐inactivated Ago2 confirmed that the response of the TO emission is not caused by a change of buffer ingredients. This method could help to estimate the functional activity of Ago2, for example for recombinant proteins, or could also be used for evaluation of the new Ago‐2 inhibitors.^20^

## Conclusion

The existing methods for evaluation of miRNA maturation are effective and allow the quantification of either pre‐miRNA or mature miRNA in *in vitro* reactions, cell lysate and cells. We demonstrated that the combined use of two, spectrally distinguishable FIT PNA probes enables the simultaneous detection of unmatured pre‐miRNA and matured miRNA molecules in unlabelled form. Given its importance as a potential target in cancer therapy we evaluated the method by monitoring the Dicer‐mediated maturation of miR21. Upon cleavage of pre‐miR21 Dicer produces a miR21‐5p▪miR21‐3p duplex and a 17‐nt hairpin (C30‐G45). A red emitting QB‐FIT PNA probe (**1**) reported the presence of pre‐miR21 by providing a 14‐fold enhancement of fluorescence. While targeting of the loop region would have been an obvious choice to discriminate between pre‐miR21 and the miR21‐5p▪miR21‐3p duplex, our data shows that probes directed against the duplex‐loop junction enable a cleavage‐specific monitoring by minimizing the turn‐on response to the C30‐G45 cleavage product.

The second, green‐emitting FIT PNA probe (**2**) provided a positive response to Dicer cleavage products by furnishing a tenfold enhancement of fluorescence upon recognition of miR21‐3p. The 7‐nt short probe was not able to invade the miR21‐5p▪miR21‐3p duplex and, therefore, monitoring of pre‐miR21 cleavage by Dicer *in vitro* was not feasible. However, this probe proved remarkably responsive to the presence of Ago2 protein, which loads the miR21‐5p strand and releases the miR21‐3p strand, thereby, making it accessible for probe hybridization. We believe that probes such as **2** can be valuable for the estimation of the functional activity of Ago2 or for the fast screening of the new Ago2 inhibitors.

Hybridization probes designed to report Dicer‐induced cleavage *in vitro* must be able to invade the miR21‐5p▪miR21‐3p duplex but not the corresponding duplex region in pre‐miR21. With the affinity‐enhanced 11‐nt PNA probe **3** we have – in principle – successfully met this goal. The TO‐FIT PNA 11mer **3** discriminates pre‐miR21 by providing a threefold higher TO emission in the presence of the miR21‐5p▪miR21‐3p duplex. Though the extent of the fluorescence response is moderate, the combined use of both QB‐FIT PNA **1** and TO‐FIT PNA **3** allowed real‐time monitoring of Dicer‐mediated miR21 maturation. In a model system, the probe set responded to cleavage by showing a tenfold increase of the TO/QB emission ratio. Experiments with rhDicer exposed the known reluctance of the recombinant enzyme to process short pre‐miRNA. The probe system uncovered a potential shortcoming of maturation experiments with rhDicer. The loss of signal for both the QB and the TO emission channels hints at further cleavage pathways when the enzyme load is high. In such case, a single probe system targeted to the mature miR21 would fail to report the enzyme's action, which highlights the advantage provided by a dual channel system including probes against both unmatured and mature miRNA molecules.

Although the probes were designed for monitoring maturation of miR21, the approach should be applicable to other miRNA systems. Targeting of junctions between duplex and hairpin regions should provide specific probes for pre‐miRNA, while targeting of the passenger miRNA strand should allow responsiveness to Ago2 loading in general. The challenge to invade the miRNA duplex formed upon Dicer cleavage can be addressed by PNA molecules. Based on this and the results shown, we are confident that PNA‐based FIT probes will prove useful for the screening of new Dicer inhibitors and pre‐miR21 binders as well as for evaluation of new targets of miR21‐3p and Ago2. In light of previous live cell RNA imaging applications with FIT probes,^15^ it may be feasible to monitor miRNA maturation in live cells.

## Experimental Section

**Solid‐phase PNA synthesis**. Automated linear solid‐phase PNA synthesis was performed on a 2 μmol scale by using an Intavis ResPep synthesizer and TentaGel R RAM resin (Rapp Polymere, Tübingen, Germany, loading 0.18 μmol/mg). Prior to the first coupling the resin was allowed to swell for 10 min in DMF in micro scale columns. For Fmoc cleavage, the resin was treated twice with DMF/piperidine (4 : 1, 0.3 mL, 2 min) and subsequently washed with DMF (6×0.3 mL). For coupling, a 0.2 M solution of Fmoc−Bhoc−PNA monomers (4 equiv, Link Technologies, Bellshill, Scotland) in NMP was mixed with. 4 equiv. HCTU and 12 equiv. of NMM in DMF and added to the resin. After 30 min the solution was discarded and the resin was washed with DMF (6x0.3 mL). Coupling of Fmoc−Aeg(TO)−OH or Fmoc−Aeg(QB)−OH was performed manually. To a 0.2 M solution of labelled PNA monomer (4 equiv) in 80 mL DMF was added 2 mg of pyridinium *p*‐toluene sulfonate, PyBOP (4 equiv) and NMM (12 equiv). The mixture was added to the resin for overnight reaction. The coupling was repeated for 2 h. After washing with DMF (5×0.5 mL), CH_2_Cl_2_ (5×0.5 mL) and DMF (5×0.5 mL) automated synthesis was continued. For capping, the resin was treated for 5 min with a solution of acetic anhydride (5 %) and 2,6*‐*lutidine (6 %) in DMF. The resin was washed by DMF (6×0.3 mL). For cleavage the vacuum dried resin was suspended in 0.5 mL cleavage mixture (95 % TFA, 2 % triisopropylsilane, 2 % H_2_O) for 1 h. The solution was collected by filtration after 1 h and cleavage repeated for 30 min. The combined filtrates were concentrated under air flow until 0.2 mL before ice cold diethyl ether (2 mL) was gently added. The precipitate formed upon 15 min incubation on dry ice was collected by centrifugation (20 min, 4000 rpm / ∼3.300 g). Preparative HPLC purification was carried out by using an Agilent 1100 Series system and a Polaris C18‐A column (250×10.0 mm) from Varian with a binary mixture of A (0.1 % TFA, 1 % acetonitrile, 98.9 % H2O) and B (0.1 % TFA, 1 % H_2_O, 98.9 % acetonitrile) as a mobile phase (flow=6.0 mL/min) in a linear gradient optimized for every PNA probe.

**Fluorescence measurements**. Fluorescence spectra were measured by using a Varian Cary Eclipse spectrometer. Each fluorescence quartz cuvette contains 150 μL of assay buffer (1× PBS:137 mM NaCl, 10 mM Phosphate, 2.7 mM KCl, and a pH of 7.4, with addition of MgCl_2_ if mentioned), 0.5 μM FIT PNA probe and 1 equiv. of RNA target (synthetic targets were purchased from BioTeZ Berlin‐Buch GmbH, Germany) or reaction product. For data analysis, buffer‐only fluorescence was subtracted.

***In vitro*** **transcription**. Pre‐miR21 was prepared by using the TranscriptAid T7 High Yield Transcription Kit (Thermo Scientific Fermentas) according to the manufacturer's protocol. T7 Primer and pre‐miR21 ssDNA template were used as a starting materials and were boiled for 3 min, 95 °C and slowly cooled down to room temperature before transcription. After in vitro transcription reaction mix was treated by DNase and follow to PCI extraction and ethanol precipitation. The pre‐miR21 DNA template tgtcagacaaaggcccatcgactggtgttgccatgagattcaacagtcaacatcagtctgataagctacccgacaccctatagtgagtcgtatta was purchased from Biomers (Ulm, Germany).

**Recombinant Dicer reaction** was performed by using the Recombinant Dicer Enzyme Kit (Genlantis, San Diego, USA) according to the manufacturer's instructions. A self‐made buffer (20 mM TRIS**⋅**HCl, pH 7.74, 2.5 mM MgCl_2_, 12 mM NaCl, 1 mM DTT) was used as described.10b For experiments described in Figure 4A, a mix comprised of pre‐miR21 in buffer and the specified amount of Dicer was incubated and added to solutions of the FIT probes after varied time points.

## Conflict of interest

The authors declare no conflict of interest.

## Supporting information

As a service to our authors and readers, this journal provides supporting information supplied by the authors. Such materials are peer reviewed and may be re‐organized for online delivery, but are not copy‐edited or typeset. Technical support issues arising from supporting information (other than missing files) should be addressed to the authors.

Supplementary
