# Supplementary material for: Monitoring Dicer‐Mediated miRNA‐21 Maturation and Ago2 Loading by a Dual‐Colour FIT PNA Probe Set
Source: Chembiochem. 2020 May 26;21(17):2527–32. doi: 10.1002/cbic.202000173 (PMC7496889; doi:10.1002/cbic.202000173)

ChemBioChem

Supporting Information

Monitoring Dicer-Mediated miRNA-21 Maturation and Ago2 Loading by a Dual-Colour FIT PNA Probe Set

Natalia Loibl, Christoph Arenz,* and Oliver Seitz*© 2020 The Authors. Published by Wiley-VCH Verlag GmbH & Co. KGaA. This is an open access article under the terms of the Creative Commons Attribution License, which permits use, distribution and reproduction in any medium, provided the original work is properly cited.

Supporting Information

1. Fluorescence properties of the pre-miR-21 specific QB PNA probes

Table S1. Fluorescence enhancements of QB-containing PNA FIT probes upon hybridization with pre-miR-21 or miR-21-5p

	I / I_0[a]	
	**addition of pre-miR-21**	**addition of miR-21-5p**
ttg-QB-catgaga	2.0	1.2
ttgc-QB-atgaga	4.9	1.0
ttgcc-QB-tgaga, **1**	13.7	1.0
ttgcca-QB-gaga	3.1	0.9
ttgccatg-QB-ga	2.7	0.9
ttgccatga-QB-a	3.5	0.9

[a] λ_{ex} = 588 nm, λ_{em}= 606 nm; Conditions: 0.5 μM probes and targets in 10 mM NaH_2PO_4, 137 mM NaCl, 2.7 mM KCl, pH 7.0, 37 °C.

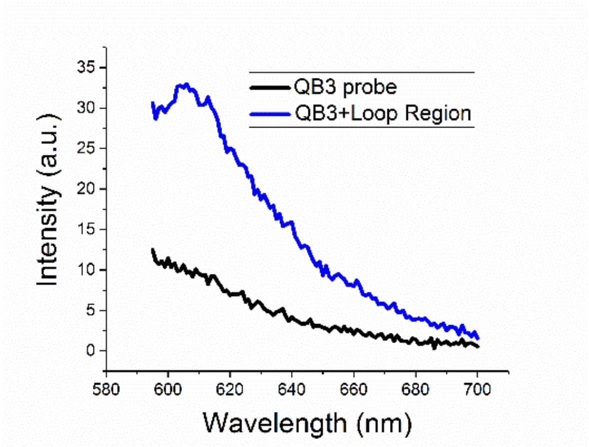

Figure S1. Fluorescence spectra of the QB-FIT PNA **1** in absence (black) and in the presence (blue) of C30-G45. Conditions: 0.5 μM probes and targets in 10 mM NaH_2PO_4, 137 mM NaCl, 2.7 mM KCl, pH 7.0, 37 °C, λ_{ex} = 588 nm.

2. Fluorescence properties of the miR-21-3p specific TO PNA probes

Table S2. Fluorescence enhancements of TO-containing PNA FIT probes upon hybridization with miR-21-3p or pre-miR-21

	I / I_0[a]	
	**addition of pre-miR-21**	**addition of miR-21-3p**
ggtg-TO-tg, **2**	5.5	9.5
ggt-TO-ttg	14.8	19.6
gg-TO-gttg	13.2	12.0
g-TO-tgttg	4.7	6.2

[a] λ_{ex} = 516 nm, λ_{em}= 536 nm; Conditions: 0.5 µM probes and targets in 10 mM NaH_2PO_4, 137 mM NaCl, 2.7 mM KCl, pH 7.0, 37 °C.

Table S3. Length variation of the miR-21-3p specific TO PNA probes and fluorescence enhancements upon hybridization with miR-21-3p or pre-miR-21 or a mix of miR-21-5p/miR-21-3p/C30-G45.

		I / I_0[a]	
	**pre-miR-21**	**miR-21-3p**	**miR-21-3p/miR-21-5p/C30-G45**
ggtg-TO-tg, **2**	3.3	10.3	4.2
tggtg-TO-tg	2.4	4.5	2.7
ctggtg-TO-tg	1.5	2.5	1.6
actggtg-TO-tg	1.1	2.1	1.5
gactggtg-TO-tg, **3**	1.7	4.4	3.4

[a] λ_{ex} = 516 nm, λ_{em}= 536 nm; Conditions: 0.5 µM probes and targets in 10 mM NaH_2PO_4, 137 mM NaCl, 2.7 mM KCl, 3 mM $MgCl_2$, pH 7.0, 37 °C.

3. UPLC-MS Data

Analysis was performed by using an Acquity system from Waters and a BEH130 C18 column (2.1 x 50 mm, 1.7 µm; heater set on 50 °C) with a binary mixture of A (0.1% TFA, 1% acetonitrile, 98.9% H2O) and B (0.1% TFA, 1% H2O, 98.9% acetonitrile) as a mobile phase (flow = 0.5 mL/min) in a linear gradient optimized for every PNA probe.

ttg-QB-catgaga

Gradient: 3 % B to 60 % B in A within 4 min: ESI-MS: m/z = 1064.6 ($(M+3H)^{3+}$, calc.: 1064.4), 798.9 ($(M+4H)^{4+}$, calc.: 798.6), Formula: $C_{135}H_{161}N_{64}O_{32}$, MW_{calc} = 3192.1598 g·mol^{-1}, Exact $Mass_{calc}$=3190.3058 g·mol^{-1}

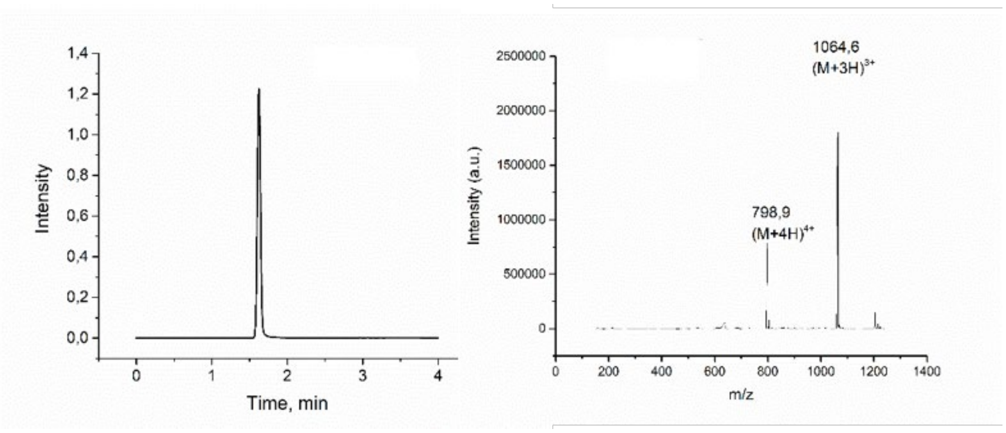

ttgc-QB-atgaga

Gradient: 3 % B to 60 % B in A within 4 min: ESI-MS: m/z = 1064.6 ($(M+3H)^{3+}$, calc.: 1064.4), 798.8 ($(M+4H)^{4+}$, calc.: 798.6), Formula: $C_{135}H_{161}N_{64}O_{32}$, MW_{calc} = 3192.1598 g·mol^{-1}, Exact $Mass_{calc}$=3190.3058 g·mol^{-1}

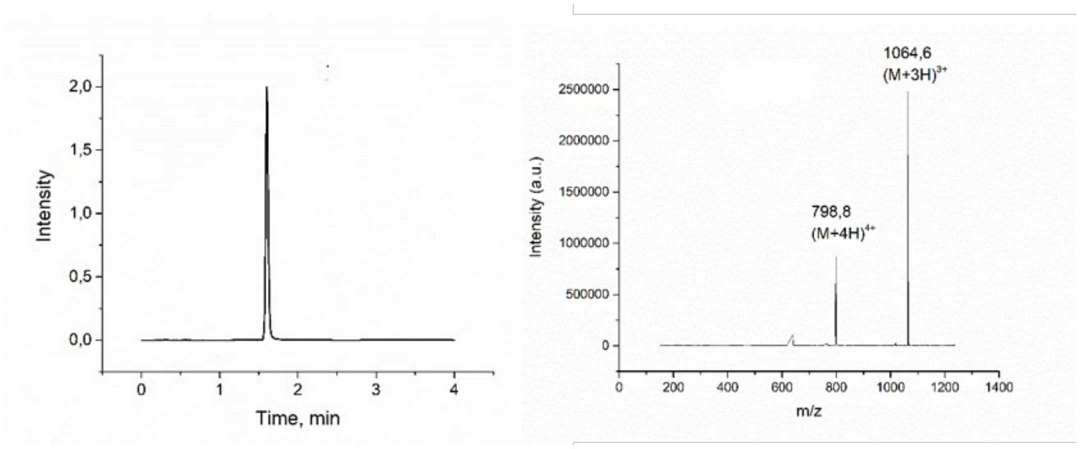

ttgcc-QB-tgaga, 1

Gradient: 3 % B to 60 % B in A within 4 min: ESI-MS: m/z = 1056.5 ($(M+3H)^{3+}$, calc.: 1056.4), 792.8 ($(M+4H)^{4+}$, calc.: 792.6), Formula: $C_{134}H_{161}N_{62}O_{33}$, MW_{calc} = 3168.1351 g·mol^{-1}, Exact $Mass_{calc}$=3166.2946 g·mol^{-1}

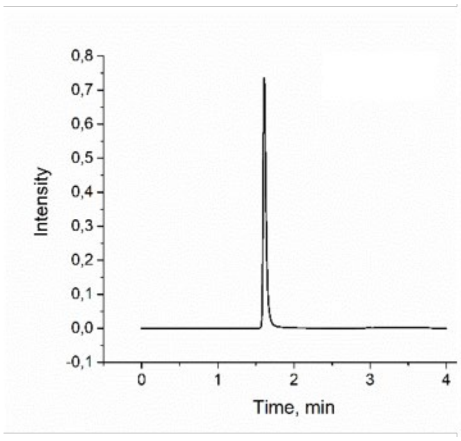

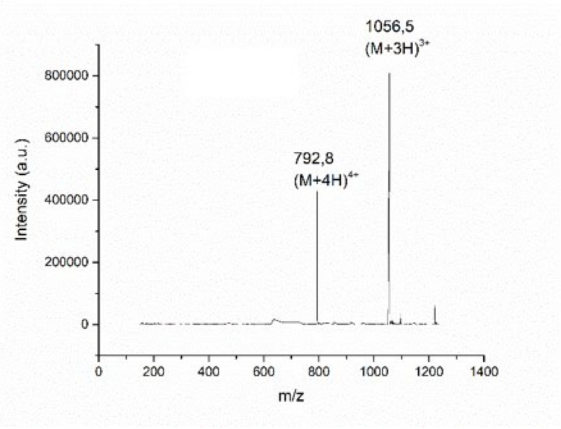

ttgcca-QB-gaga

Gradient: 3 % B to 60 % B in A within 4 min: ESI-MS: m/z = 1059.6 ($(M+3H)^{3+}$, calc.: 1059.4), 795.1 ($(M+4H)^{4+}$, calc.: 794.8), Formula: $C_{134}H_{160}N_{65}O_{31}$, MW_{calc} = 3177.1485 g·mol^{-1}, Exact $Mass_{calc}$=3175.3061 g·mol^{-1}

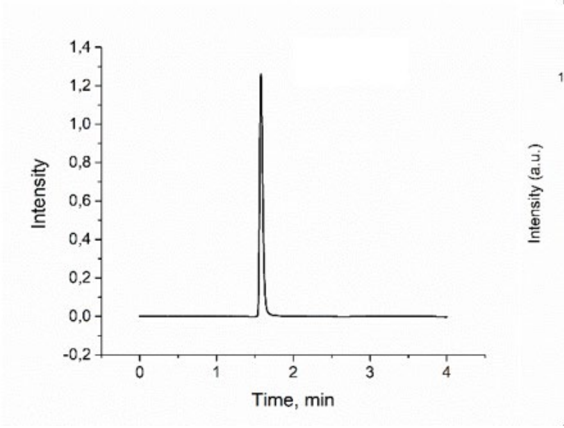

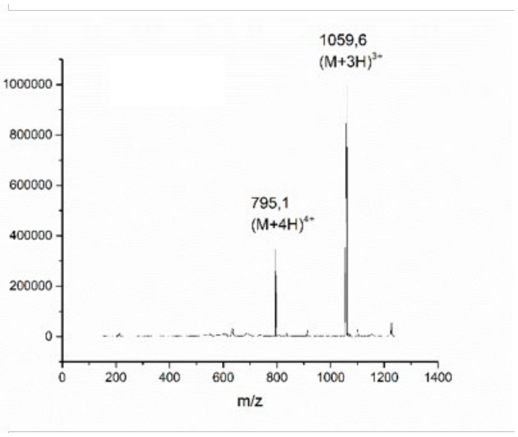

ttgccatg-QB-ga

Gradient: 3 % B to 60 % B in A within 4 min: ESI-MS: m/z = 1056.6 ($(M+3H)^{3+}$, calc.: 1056.4), 792.9 ($(M+4H)^{4+}$, calc.: 792.6), 634.6 ($(M+5H)^{5+}$, calc.: 634,3), Formula: $C_{134}H_{161}N_{62}O_{33}$, MW_{calc} = 3168.1351 g·mol^{-1}, Exact $Mass_{calc}$=3166.2946 g·mol^{-1}

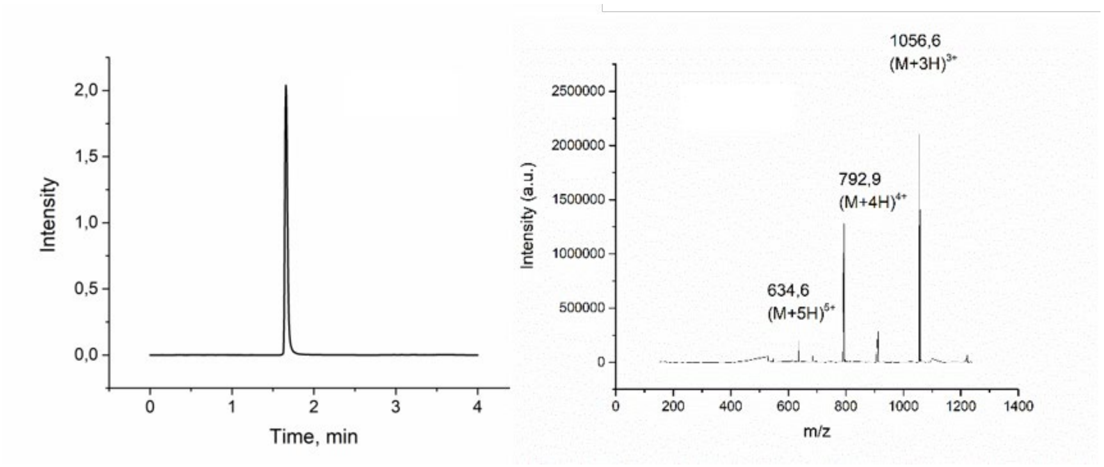

ttgccatga-QB-a

Gradient: 3 % B to 60 % B in A within 4 min: ESI-MS: m/z = 1051.2 ($(M+3H)^{3+}$, calc.: 1051.1), 788.9 ($(M+4H)^{4+}$, calc.: 788.6), Formula: $C_{134}H_{161}N_{62}O_{32}$, MW_{calc} = 3152.1357 g·mol^{-1}, Exact $Mass_{calc}$=3150.2996 g·mol^{-1}

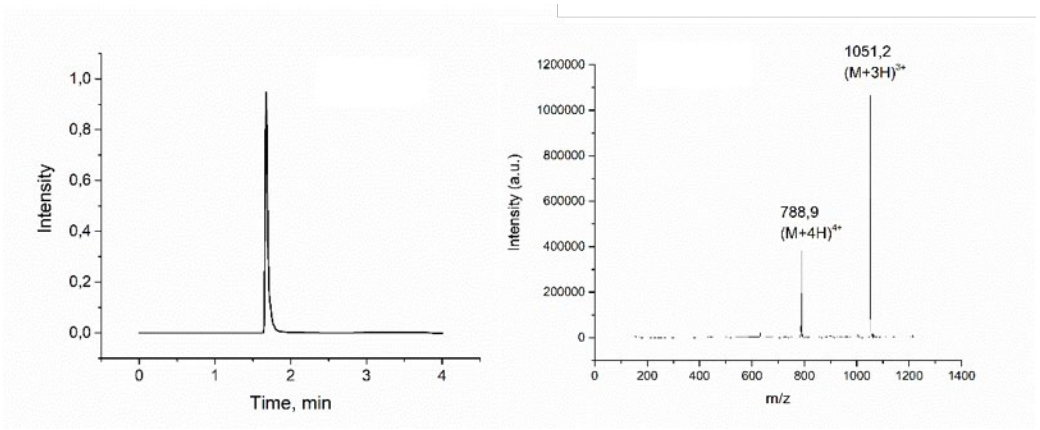

ggtg-TO-tg, 2

Gradient: 3 % B to 60 % B in A within 4 min: ESI-MS: m/z = 1073.4 ($(M+2H)^{2+}$, calc.: 1073.4), 716.1 ($(M+3H)^{3+}$, calc.: 715.9), Formula: $C_{90}H_{106}N_{41}O_{22}S_1$, MW_{calc} = 2146.1509 g·mol^{-1}, Exact $Mass_{calc}$=2144.8215 g·mol^{-1}

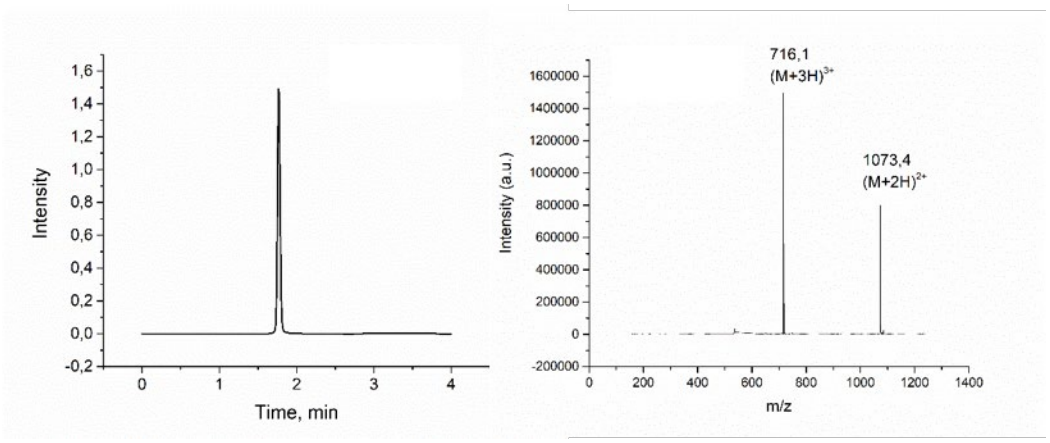

ggt-TO-ttg

Gradient: 3 % B to 60 % B in A within 4 min: ESI-MS: m/z = 1060.9 ($(M+2H)^{2+}$, calc.: 1060.9), 707.7 ($(M+3H)^{3+}$, calc.: 707.6), 531.0 ($(M+4H)^{4+}$, calc.: 531.0), Formula: $C_{90}H_{107}N_{32}O_{23}S_1$, MW_{calc} = 2121.1381 g·mol^{-1}, Exact $Mass_{calc}$=2119.815 g·mol^{-1}

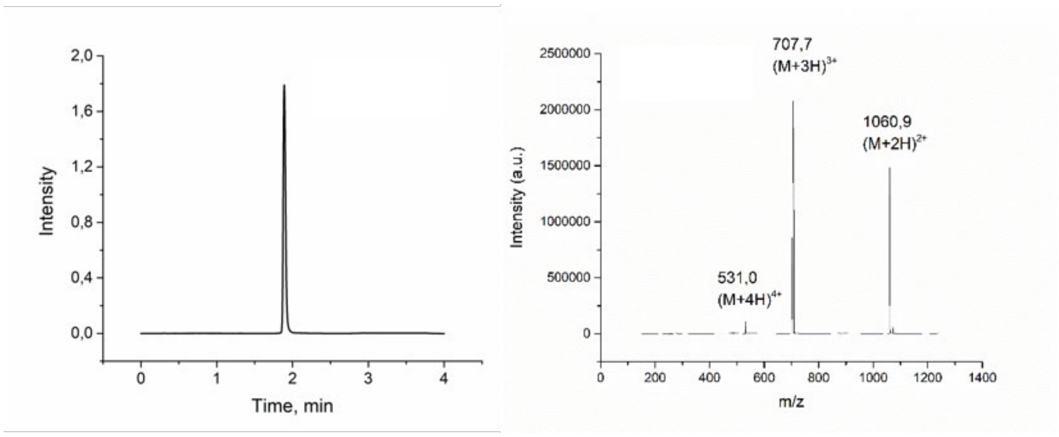

gg-TO-gttg

Gradient: 3 % B to 60 % B in A within 4 min: ESI-MS: m/z = 1073.3 ((M+2H)$^{2+}$, calc.: 1073.4), 715.8 ((M+3H)$^{3+}$, calc.: 715.9), Formula: $C_{90}H_{106}N_{41}O_{22}S_1$, MW_{calc} = 2146.1509 g·mol^{-1}, Exact $Mass_{calc}$=2144.8215 g·mol^{-1}

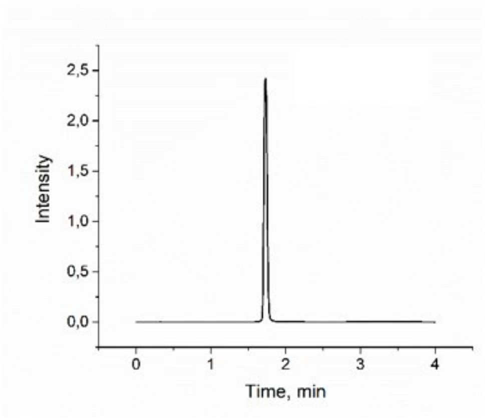

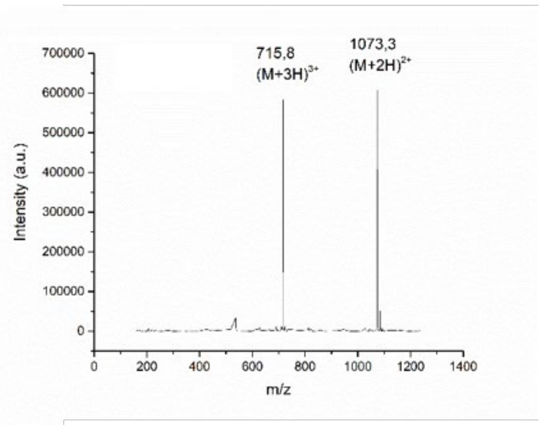

g-TO-tgttg

Gradient: 3 % B to 60 % B in A within 4 min: ESI-MS: m/z = 1061.0 ((M+2H)$^{2+}$, calc.: 1060.9), 707.8 ((M+3H)$^{3+}$, calc.: 707.6), Formula: $C_{90}H_{107}N_{32}O_{23}S_1$, MW_{calc} = 2121.1381 g·mol^{-1}, Exact $Mass_{calc}$=2119.815 g·mol^{-1}

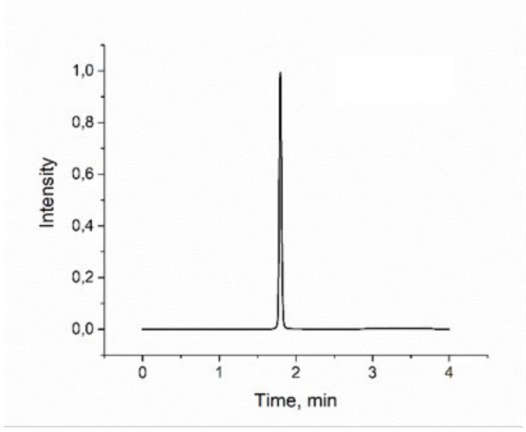

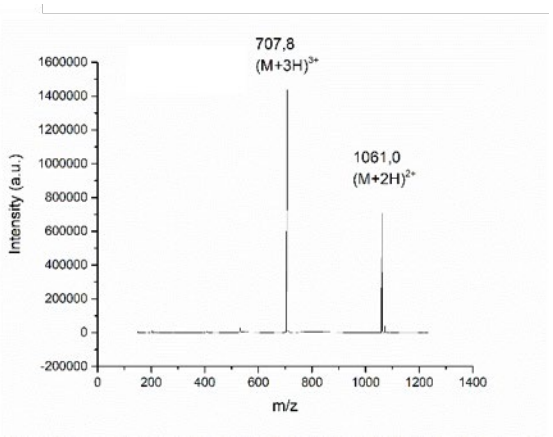

tggtg-TO-tg

Gradient: 3 % B to 60 % B in A within 4 min: ESI-MS: m/z = 1206.6 ($(M+2H)^{2+}$, calc.: 1206.5), 804.7 ($(M+3H)^{3+}$, calc.: 804.6), 603.8 ($(M+4H)^{4+}$, calc.: 603.7), Formula: $C_{101}H_{120}N_{45}O_{26}S_1$, MW_{calc} = 2412.4041 g·mol^{-1}, Exact $Mass_{calc}$=2410.923 g·mol^{-1}

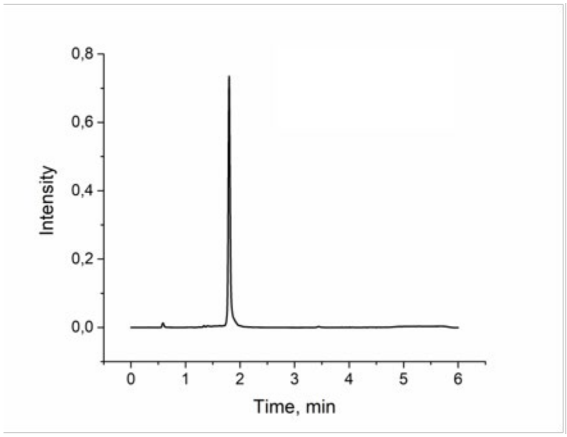

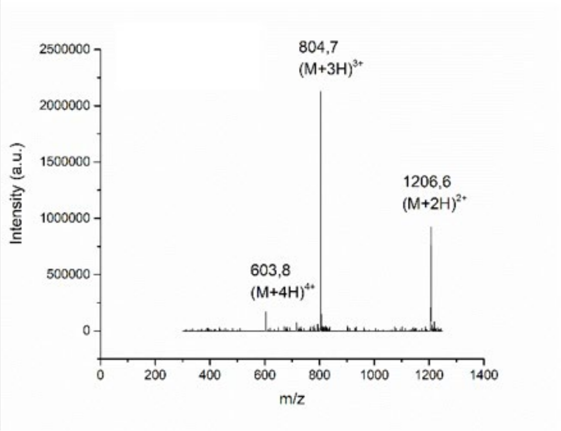

ctggtg-TO-tg

Gradient: 3 % B to 60 % B in A within 4 min: ESI-MS: m/z = 888.7 ($(M+3H)^{3+}$, calc.: 888.3), 667.1 ($(M+4H)^{4+}$, calc.: 666.5), Formula: $C_{111}H_{133}N_{50}O_{29}S_1$, MW_{calc} = 2663.646 g·mol^{-1}, Exact $Mass_{calc}$=2662.0248 g·mol^{-1}

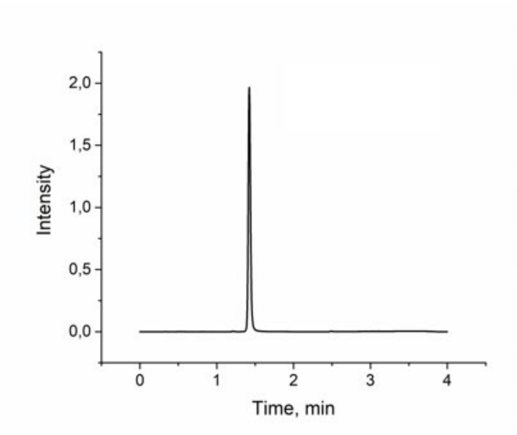

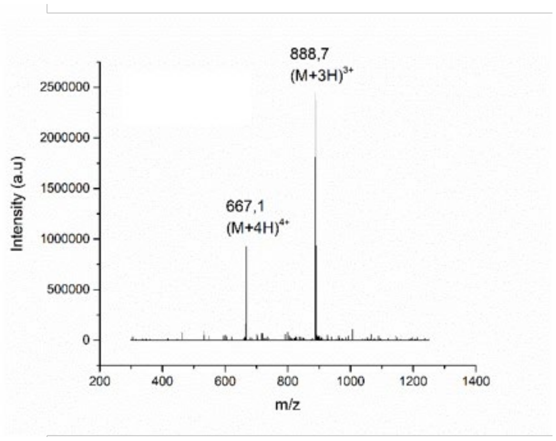

actggtg-TO-tg

Gradient: 3 % B to 60 % B in A within 4 min: ESI-MS: m/z = 980.2 ((M+3H)$^{3+}$, calc.: 980.1), 735.4 ((M+4H)$^{4+}$, calc.: 735.3), 588.6 ((M+5H)$^{5+}$, calc.: 588.4) Formula: $C_{122}H_{146}N_{57}O_{31}S_1$, MW_{calc} = 2938.9126 g·mol^{-1}, Exact $Mass_{calc}$=2937.1378 g·mol^{-1}

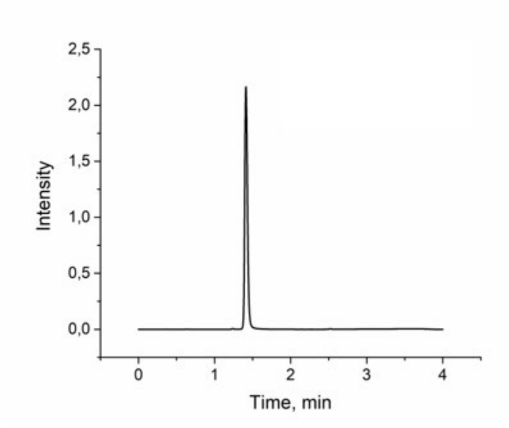

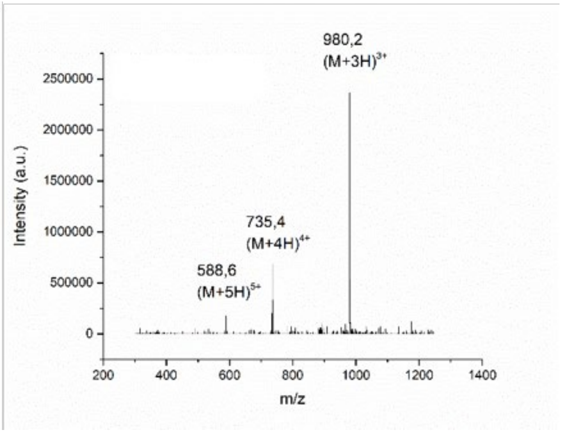

gactggtg-TO-tg, 3

Gradient: 3 % B to 60 % B in A within 4 min: ESI-MS: m/z = 1077.4 ((M+3H)$^{3+}$, calc.: 1077.1), 808.2 ((M+4H)$^{4+}$, calc.: 808.1), 646.2 ((M+5H)$^{5+}$, calc.: 646.7) Formula: $C_{133}H_{159}N_{64}O_{34}S_1$, MW_{calc} = 3230.1786 g·mol^{-1}, Exact $Mass_{calc}$=3228.2458 g·mol^{-1}

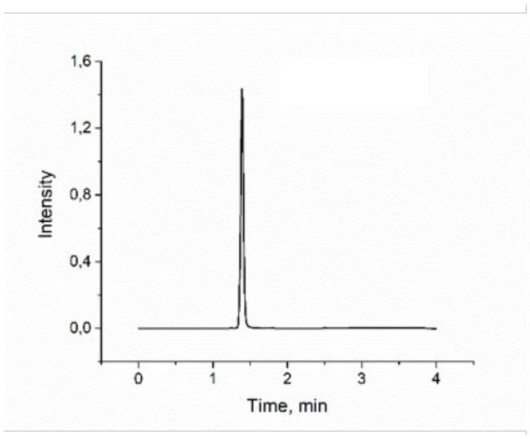

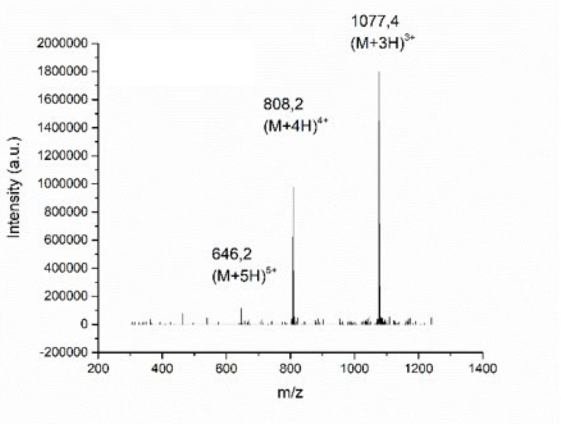

Supplement: Supplementary file 1 — Supplementary [file CBIC-21-2527-s001.pdf]
